# Energy expenditure (EE) in mechanically ventilated patients: espen equation using different body weights (BW) vs. indirect calorimetry (IC)

**DOI:** 10.1186/2197-425X-3-S1-A293

**Published:** 2015-10-01

**Authors:** S Graf, L Genton, T Oshima, C Pichard, CP Heidegger

**Affiliations:** Adult Intensive Care, Geneva University Hospital, Geneva, Switzerland; Nutrition Unit, Geneva University Hospital, Geneva, Switzerland; Hopitaux Universitaires de Geneve, Geneve, 14 Switzerland

## Introduction

IC is the reference method to measure EE, but is not available in every healthcare center. ESPEN guidelines recommend using a predictive equation based on the BW before acute illness in case of fluid retention, but the accuracy of this BW is questionable.

## Objectives

The aim of this study is to ascertain the accuracy of the ESPEN equation using different BW vs. EE measured by IC, in ventilated patients and to determine the most suitable BW.

## Methods

All mechanically ventilated patients staying >72h in ICU, with Fi02 < 60%, PEEP < 9cmH_2_O, no pulmonary fistula or lung multi-resistant bacteria were included and had IC measurement. We calculated EE with the ESPEN equation (20-25 kcal/kg acute phase and 25-30 kcal/kg post-acute phase), using several BW: anamnestic (BW_AN_), measured (BW_MES_), adjusted for water balance (BW_ADJ_) and ideal BW calculated for a body mass index of 22.5 and 25 kg/m^2^. Results are presented as mean ± SD. Calculated EE was compared to EE measured by IC, with ANOVA repeated measure and Bonferroni post-hoc test, as well as Bland-Altman analysis.

## Results

We included 85 patients (57 ± 19 y, 61 men, SAPS II 50 ± 14), including 47 in acute phase. EE calculated with BW_AN_, BW_ADJ_ and BW_MES_ significantly overestimated measured EE by IC (1910 ± 458 kcal/d; p < 0.0001). Differences of calculated EE were statistically significant between the various BW used (p < 0.0001).

**Table 1:** Differences of EE according to various BW.

ESPEN equation	Mean Δ (calculated- measured EE)( ± 2SD)*p < 0.05 Bonferroni post hoc test
BWAN	271 (-1273 ; 731)*
BWADJ	265 (-1339 ; 809)*
BWMES	368 (-1478 ; 742)*
BWBMI22.5	-15 (-811 ; 841)
BWBMI25	195 (-1041 ; 651)

## Conclusions

It is crucial to define the best BW to be used because it impacts calculated EE. The ideal BW calculated for a BMI at 22.5 kg/m^2^ matches better with measured EE than other BW. ESPEN equation is not accurate enough to be used in a metabolically heterogeneous ICU population. It tends to overestimate the EE increasing the risk of overnutrition and potential negative impact on clinical outcome.

